# Is pregnancy loss initiated by embryonic death or luteal regression? Profiles of pregnancy-associated glycoproteins during elevated progesterone and pregnancy loss

**DOI:** 10.3168/jdsc.2022-0282

**Published:** 2022-12-22

**Authors:** Rafael R. Domingues, Joao Paulo N. Andrade, Thiago O. Cunha, Guilherme Madureira, Uzi Moallem, Victor Gomez-Leon, Joao Paulo N. Martins, Milo C. Wiltbank

**Affiliations:** 1 Department of Animal and Dairy Sciences, University of Wisconsin-Madison, Madison 53706; 2 Endocrinology and Reproductive Physiology Program, University of Wisconsin-Madison, Madison 53706; 3 Department of Ruminant Science, Institute of Animal Sciences, Volcani Institute, Rishon LeZion, Israel 50250; 4 Department of Animal Sciences and Industry, Kansas State University, Manhattan 66506; 5 Department of Medical Sciences, School of Veterinary Medicine, University of Wisconsin-Madison, Madison 53706

## Abstract

Because progesterone (P4) is essential for pregnancy establishment and maintenance, we investigated the effect of increased concentrations of P4 on embryonic attachment and concentrations of pregnancy-associated glycoproteins (PAG). Additionally, we investigated the relationships among luteal regression, pregnancy loss, and PAG concentrations in cows undergoing pregnancy loss by d 33 of pregnancy. Lactating dairy cows were allocated into control (n = 40) and human chorionic gonadotropin (hCG; 3,300 IU on d 7 and 13 to promote greater circulating P4; GnRH = d 0; n = 46) groups. Progesterone was measured daily from d 7 to 33, and PAG was measured daily from d 17 to 33; both hormones were also measured on d 47 and 61. An increase in PAG >10% compared with d 17 was considered a marker for pregnancy. The gold standard for pregnancy diagnosis was ultrasound evaluation of embryonic heartbeat on d 33. Statistical analyses were done with PROC MIXED from SAS Institute Inc. Concentrations of P4 were greater from d 8 onward in the hCG group. Concentrations of PAG did not differ between groups from d 17 to 33, suggesting no effect of increased P4 on hastening embryonic attachment and placental development. Nevertheless, PAG was greater in the hCG group on d 47 and 61, suggesting greater placental area or PAG secretory capacity. Pregnancy loss between d 20 and 33 occurred in 24.6% of cows. About 50% of pregnancy loss was due to luteal regression and about 50% was due to conceptus failure; that is, a decrease in PAG in the absence of luteal regression. In conclusion, increased P4 does not hasten embryonic attachment or early placental development but it leads to increased PAG in the second half of the second month of gestation. Additionally, pregnancy loss seems to be initiated by either corpus luteum regression or conceptus failure.

## Highlights

• Increased P4 did not hasten embryonic attachment, measured by first PAG increase.
• Increased P4 increased PAG during second half of the second month of pregnancy.
• About 50% of pregnancy loss between d 20 and 33 was initiated by luteal regression.
• About 50% of pregnancy loss between d 20 and 33 was initiated by conceptus failure.

## Graphical Abstract

**Figure:**
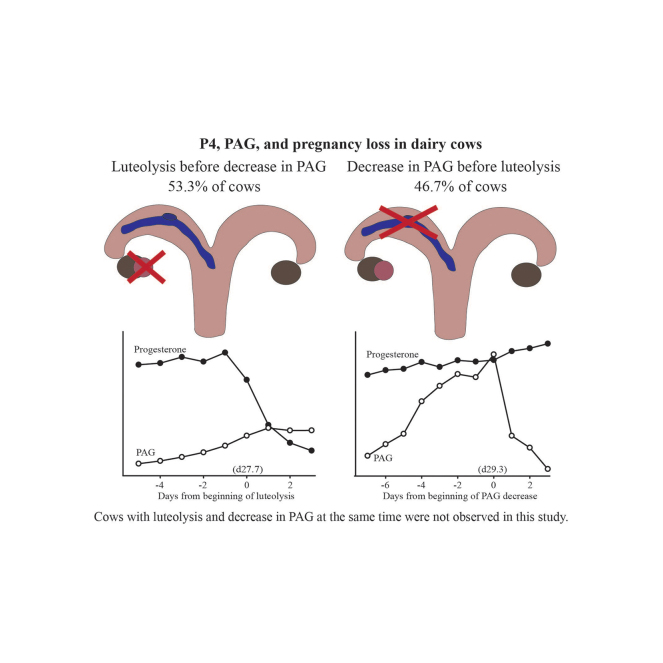
**Summary:** We investigated the effect of increasing circulating progesterone (P4) on timing of embryonic attachment as measured by daily pregnancy-associated glycoproteins (PAG) concentrations. Further, to investigate the underlying cause of pregnancy loss, the timing of corpus luteum (CL) regression and embryonic death was determined by daily P4 and PAG in dairy cows undergoing pregnancy loss. Until d 33, PAG was similar, regardless of elevated P4 concentrations, but it was greater on d 47 and d 61 of pregnancy in cows with greater P4. Pregnancy loss between d 20 and 33 could be initiated by either CL regression (P4 decrease, constant PAG; 53% of cows) or embryonic death (PAG decrease, constant P4; 47% of cows).

The endocrine function of the corpus luteum (**CL**) in maintaining pregnancy was first recognized in the early 1900s (Fraenkel and Cohn, 1901; Magnus, 1901), and it is now clear that circulating progesterone (**P4**) from the CL is the critical hormone essential for pregnancy maintenance and many other aspects of female reproduction (Forde et al., 2009; Wiltbank et al., 2014). Because of the critical role of P4 in embryonic survival and development, multiple studies have manipulated P4 concentrations during early gestation aiming to understand the effect of P4 concentrations on pregnancy rates and pregnancy loss and to improve reproductive efficiency in cattle (Clemente et al., 2009; García-Guerra et al., 2020). For example, increasing circulating concentrations of P4 in heifers by insertion of an intravaginal P4 device on d 3 of the estrous cycle increased embryo elongation and area by d 14 (Clemente et al., 2009). In addition, induction of an accessory CL by treatment with GnRH or human chorionic gonadotropin (**hCG**) on d 5 to 7 of the estrous cycle decreased pregnancy loss in embryo transfer recipients (Niles et al., 2019; García-Guerra et al., 2020). Nevertheless, mixed results on fertility have been obtained from studies that elevated P4 concentrations after insemination in lactating dairy cows, depending on the method of P4 supplementation and timing of treatments (Monteiro et al., 2015, 2021; Sánchez et al., 2018).

In cattle, the embryo enters the uterus around 5 d post-ovulation and stays in a free-floating state for about 2 wk until attachment to the uterine endometrium (Spencer, 2013; Wiltbank et al., 2016a). Near d 21 post-ovulation, embryonic attachment and early placentation occur. The ruminant placenta is unique in that chorionic cells of embryonic origin and uterine endometrial cells fuse, forming bi- or multinucleated giant cells characteristic of the synepitheliochorial placenta of cattle (Bazer et al., 2011). These multinucleated cells synthesize pregnancy-associated glycoproteins (**PAG**), which have been used as a marker of attachment, early placentation, and pregnancy establishment in cattle for research and as a method for pregnancy diagnosis (Sasser et al., 1986; Green et al., 2005; Middleton and Pursley, 2019). Multiple PAG have been reported to date, although the first one described, PAG1 [also known as pregnancy-specific protein B (PSPB) but defined as PAG in this article], remains one of the most studied and used in clinical practice. Unfortunately, the specific physiologic role of PAGs in ruminant reproduction remains unknown.

Pregnancy loss in lactating dairy cows can occur at multiple stages of gestation, with elevated incidence (20–50%) during the first week after insemination due to fertilization failure and early embryonic loss and a further ~40% pregnancy loss occurring between d 8 and 32 of gestation (Santos et al., 2004; Wiltbank et al., 2016b). From d 8 to 32, the conceptus undergoes tremendous changes, hatching from the zona pellucida, changing shape from a blastocyst to an elongated embryo, secreting IFN-τ as the key signal for luteal maintenance, attaching to the uterine endometrium, and beginning development of the placenta. The embryonic source of nutrients and growth factors also switches from direct histotrophic nutrition to a choriovitelline type of nutrition, and finally to a chorioallantoic placenta, placentomes, with nutrition provided from maternal to fetal bloodstream (Bazer et al., 2011; Wiltbank et al., 2016b). During this critical period for pregnancy establishment in cattle, the temporal relationship between luteal regression, embryonic maintenance/loss, and placental development is not sufficiently defined.

Based on the concept that increased P4 concentrations may result in increased embryonic elongation and thereby earlier attachment and placentation, we aimed to investigate the effect of P4 concentrations on systemic concentrations of PAG as a measure of embryonic development, attachment, and early placental development. We hypothesized that greater concentrations of P4 are associated with earlier attachment and greater PAG concentrations. Additionally, we investigated the relationship between luteal regression, pregnancy loss, and PAG concentrations in cows undergoing pregnancy loss by d 33 of pregnancy. We hypothesized that pregnancy loss in high-producing dairy cows occurs in some cows due to luteal failure and in other cows due to failure of the conceptus before luteal regression.

All experimental procedures and animal management were approved by the Animal Care and Use Committee of the College of Agriculture and Life Sciences at the University of Wisconsin-Madison (Protocol ID: A006164).

The experiment was conducted at Emmons Blaine Dairy Cattle Center from the University of Wisconsin-Madison. Lactating dairy cows submitted for timed AI (**TAI**) between May and July were included in the study. Cows received a Double-Ovsynch synchronization protocol or an ovulation resynchronization protocol (Ovsynch). The day of final GnRH treatment of the protocol was considered d 0, and all cows received TAI approximately 16 h later. Only cows that ovulated by d 3 were included in the study. Cows were randomly allocated according to parity into 1 of 2 groups: control (n = 40) or human chorionic gonadotropin (**hCG**; n = 46). Cows in the hCG group received intramuscular hCG treatment (3,300 IU) on d 7 and 13 post-GnRH. The control group received the same volume of saline.

Blood samples were collected, and rectal ultrasonography was performed daily from d 6 until 33 and on d 47 and 61. Blood was collected into tubes containing EDTA by venipuncture of coccygeal vessels and put on ice until centrifugation. Plasma was isolated using centrifugation at 1,800 × *g*, decanted, and stored at –20°C until assayed for P4 and PAG. Concentrations of P4 were determined by solid-phase RIA kit containing antibody-coated tubes and ^125^I-labeled P4 (ImmuChem Coated Tube P4 ^125^I RIA Kit, MP Biomedicals) as described and validated in our laboratory (Melo et al., 2018). The sensitivity, intraassay coefficient of variation (**CV**), and interassay CV were 0.1 ng/mL, 5.5%, and 6.1%, respectively. Concentrations of PAG were determined by ELISA (BioPRYN Flex; BioTracking LLC). The intra- and interassay CV were 3.0 and 4.2%, respectively. Visualization of embryonic heartbeat on d 33 was the gold standard for pregnancy diagnosis. A 10% increase in concentration of PAG after d 17 was indicative of embryonic attachment as described previously (Middleton and Pursley, 2019). Pregnancy loss was determined by a 10% increase in PAG concentrations or ultrasound visualization of an embryo between d 28 and 32 followed by lack of embryonic heartbeat on d 33. The day of luteolysis was the day before a 50% decrease in P4 concentration from the average of the 3 highest values (Domingues et al., 2020).

All statistical analyses were performed with SAS (version 9.4; SAS Institute Inc.); PROC MIXED procedure was used, and the Tukey honestly significant difference (HSD) post hoc test was performed to determine difference between means. There were no effect of parity or sire on P4 or PAG concentrations; therefore, they were removed from the model. The final working model included only group, time, and a group × time interaction. A probability ≤0.05 indicated a difference was significant, and a probability between >0.05 and ≤0.1 indicated that significance was approached. Data are presented as the mean ± standard error of mean (SEM), unless otherwise indicated.

Ovarian dynamics, including ovulation rate to hCG treatments on d 7 and 13, total number of CL, and the effect of hCG treatment on ovarian function and timing of luteolysis and subsequent ovulation will be addressed elsewhere. Pregnancy per AI on d 33 tended to be less in the hCG group than in the control group [43.5% (20/46) vs. 65% (26/40), respectively; *P* = 0.054] but did not differ between groups on d 61 [55% (22/40) control vs. 39.1% (18/46) hCG; *P* = 0.19]. Nevertheless, the present study was not designed to investigate the effect of hCG treatment on pregnancy per AI or pregnancy loss. Rather, it was designed to investigate the effect of P4 concentrations on conceptus attachment and the relationship between P4, PAG, and pregnancy loss in high-producing dairy cows.

In pregnant cows, concentrations of P4 were greater in the hCG group from d 8 through 61 (Figure 1). As shown previously, treatment with ovulatory doses of either hCG or GnRH during the luteal phase can promote ovulation of a dominant follicle(s) (if present) within 24 to 32 h, resulting in increased circulating concentrations of P4 due to the presence of accessory CL(s) (Santos et al., 2001; Niles et al., 2019; García-Guerra et al., 2020; Monteiro et al., 2021). Nevertheless, hCG also stimulates P4 synthesis from the original CL, as evidenced by the increase in P4 concentrations by d 8 in the present study (i.e., before development of accessory CL) and the increase in luteal volume by 2 d after treatment as previously shown (Sánchez et al., 2018; Cunha et al., 2022). Additionally, hCG treatment on d 13 caused a further increase (73.6%) in P4 concentrations by d 15, suggesting additional stimulation by the second hCG treatment on P4 synthesis by the original CL or the accessory CL(s), resulting in 139.4% greater P4 concentrations in the hCG group overall from d 15 to 33 compared with the control group.

**Figure 1:**
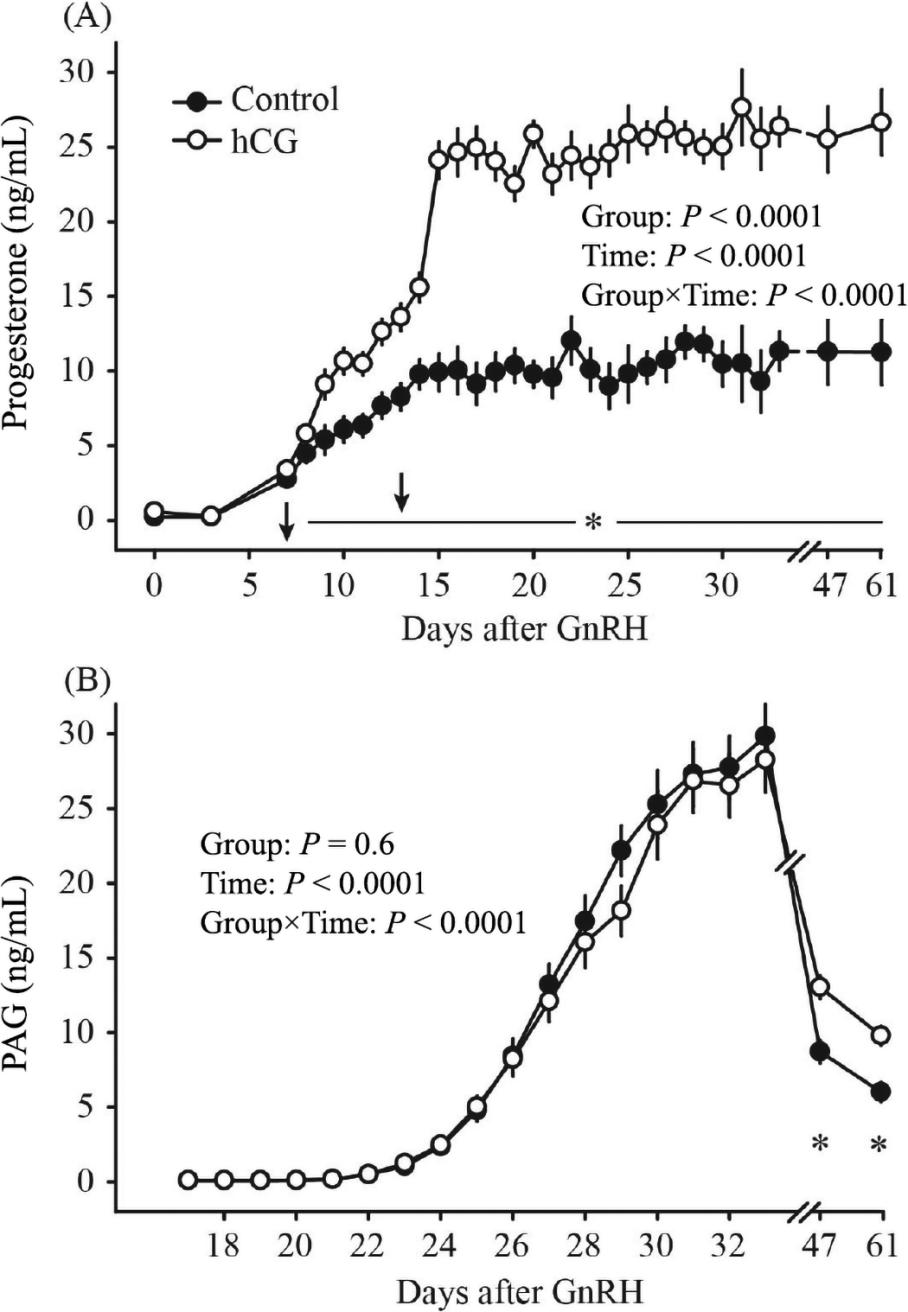
Concentrations of (A) progesterone and (B) pregnancy-associated glycoproteins (PAG) in lactating cows n = 26 for control; n = 20 for hCG). The hCG group received 3,300 IU of human chorionic gonadotropin (hCG) on d 7 and d 13 after GnRH (arrow). *Indicates significant difference between groups.

For concentrations of PAG in pregnant cows (no pregnancy loss by d 61), the main effect of time and the group × time interaction were significant (Figure 1). From d 17 until 33, concentrations of PAG were not different between control and hCG groups. After d 33, PAG decreased in both groups, similar to previous reports of greater PAG at the end of the first month of gestation compared with the second half of the second month of gestation (Green et al., 2005; Giordano et al., 2012). Nevertheless, PAG was greater in the hCG group on d 47 and 61 in the present study. On a similar note, PAG concentrations tended to be greater on d 46 and 60 and were greater on d 80 when comparing cows with P4 concentrations above versus below the average P4 concentrations (Ayad et al., 2007). In nonpregnant cows, concentrations of PAG did not significantly increase above baseline (d 17; data not shown). Compared with PAG concentrations on d 17, the first significant increase in pregnant cows occurred on d 21 for both control and hCG groups (0.10 ± 0.02 on d 17 vs. 0.18 ± 0.03 ng/mL on d 21 combined for control and hCG groups; *P* = 0.024). Similarly, the first day of significance difference in PAG concentrations between pregnant and nonpregnant cows (based on ultrasound visualization of an embryo heartbeat on d 33) was d 21 (0.18 ± 0.03 vs. 0.07 ± 0.02 ng/mL, respectively, combined for control and hCG groups; *P* = 0.023).

In a recent study (Middleton and Pursley, 2019), a 10% increase in PAG concentrations between d 17 and 24 post-AI in an individual cow resulted in 100% sensitivity for identifying nonpregnant cows; that is, if concentrations of PAG do not increase above 10% by d 24, the cow is not pregnant. In the present study, the day of a 10% increase in concentration of PAG from the baseline value on d 17 did not differ (*P* = 0.9) between control and hCG groups (d 21.3 ± 0.2 vs. d 21.3 ± 0.3, respectively). The increase in PAG concentrations by d 21 suggests that bi- or multinucleated giant cells are being formed and beginning to synthesize PAG, indicating initial development of the placenta. It is intriguing that maximal expression of IFN-τ (~d 21) and subsequent rapid downregulation of IFN-τ gene expression coincides with initial attachment, development of bi- or multinucleated cells, and secretion of PAG, as previously suggested (Ezashi and Imakawa, 2017; Imakawa et al., 2017). Although many of the transcriptional mechanisms that downregulate IFN-τ have been described, the biological relevance of the temporal association between formation of bi- or multinucleated cells and cessation of IFN-τ expression remains to be elucidated.

Our hypothesis that greater concentrations of P4 are associated with earlier embryonic attachment was not supported. Although this concept is not new (Wiltbank et al., 1961), recent studies have attempted to increase P4 to enhance embryo development and pregnancy rates (Clemente et al., 2009; Monteiro et al., 2015). This is the first study demonstrating that the timing of embryonic attachment was not different for cows with increased P4 concentrations. The lack of an effect of greater P4 on the timing of embryonic attachment may be explained as follows: (1) the increase in P4 was not early enough to modulate conceptus development or attachment, (2) the increase in P4 did not modulate uterine receptivity for attachment, or (3) greater P4 concentrations do not hasten embryonic attachment. Nevertheless, the second aspect of our hypothesis—that greater P4 would increase PAG production—was partially supported, with greater P4 resulting in greater concentrations of PAG on d 47 and 61 but not at earlier times. The biological meaning of greater PAG during the second month of gestation is unknown. Indeed, the role of placental PAG in cattle remains to be elucidated. Yet, greater PAG concentrations indicated that the elevated P4 either increased the number of placental PAG-secreting cells or increased placental synthesis of PAG per cell. Because cows with twin pregnancy (i.e., greater placental area) have greater PAG concentrations than cows with a singleton pregnancy, it seems likely that P4 increased placental area, resulting in greater systemic concentrations of PAG (Giordano et al., 2012). Further studies are needed to unravel the role of PAG on pregnancy maintenance in cattle.

Pregnancy loss between d 20 (earliest increase in PAG above 10%) and d 33 occurred in 24.6% of cows (15/61; 5 control and 10 hCG). For cows undergoing pregnancy loss, the mean day of 10% increase in PAG concentrations occurred later (*P* = 0.0006) compared with cows that remained pregnant (d 22.8 ± 0.6 vs. d 21.3 ± 0.1, respectively, combined for control and hCG groups because there was no group difference for PAG by d 33), and maximal PAG occurred on d 29.4 ± 1.0 (range d 22 to 33). From d 22 onward, concentrations of PAG were lower (*P* < 0.0001) in cows that had pregnancy loss than in cows that maintained pregnancy (not shown). The individual profile of PAG for all cows (n = 15) that had pregnancy loss before d 33 is shown in Figure 2.

**Figure 2:**
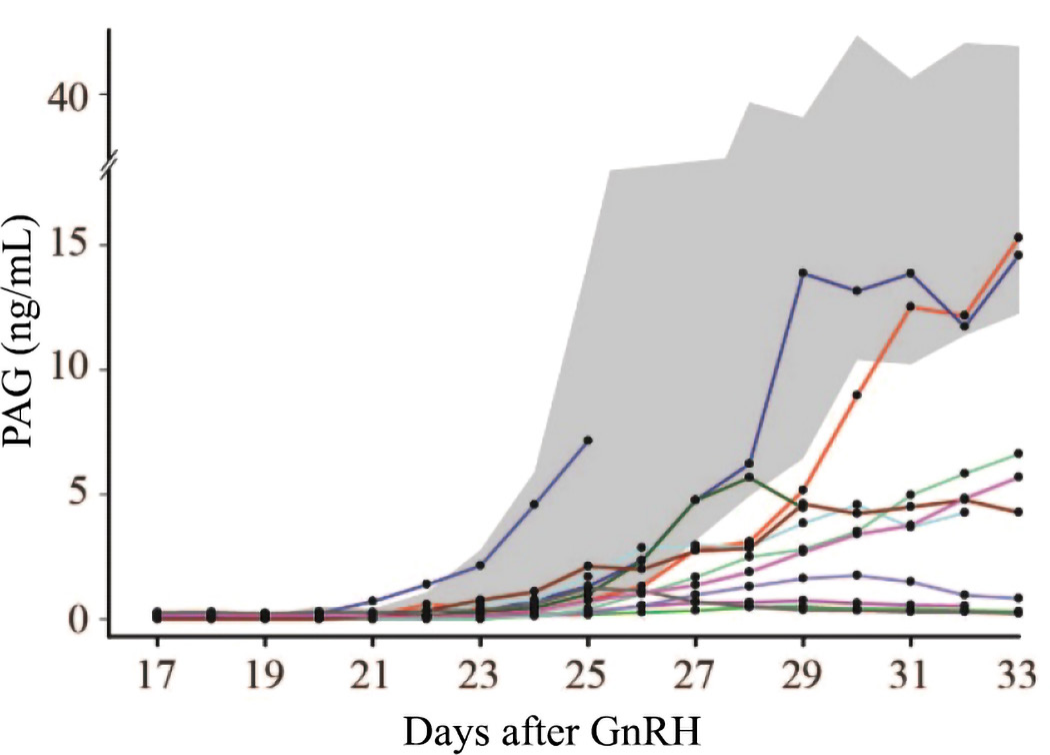
Concentrations of pregnancy-associated glycoproteins (PAG) for each cow undergoing pregnancy loss between d 20 and 33 (n = 15). Concentrations of PAG were measured daily from d 17 until 33; cows with incomplete data ovulated before d 33. The shaded area depicts the range of PAG for cows that maintained pregnancy.

To further explore the relationship between P4 and pregnancy loss, we investigated the temporal association of P4 and PAG concentrations in cows undergoing pregnancy loss before d 33 (Figure 3). In 53.3% (8/15) of cows, luteolysis occurred before (4.0 ± 1.5 d) any decrease in concentrations of PAG, suggesting that pregnancy loss was initiated by CL regression and a failure to maintain adequate P4 concentrations to maintain gestation. The mean day of luteolysis was d 27.7 ± 1.5 (range d 21 to 32). Conversely, in 46.7% (7/15) of cows with pregnancy loss between d 20 and 33, concentrations of PAG began to decrease before luteolysis (luteolysis occurred on d 32 in 1 cow and was not initiated by d 33 in 6 cows), suggesting failure of the conceptus rather than maternal failure to maintain adequate P4 concentrations. The mean day for the beginning of the decrease in PAG concentrations was d 29.3 ± 1.5.

**Figure 3:**
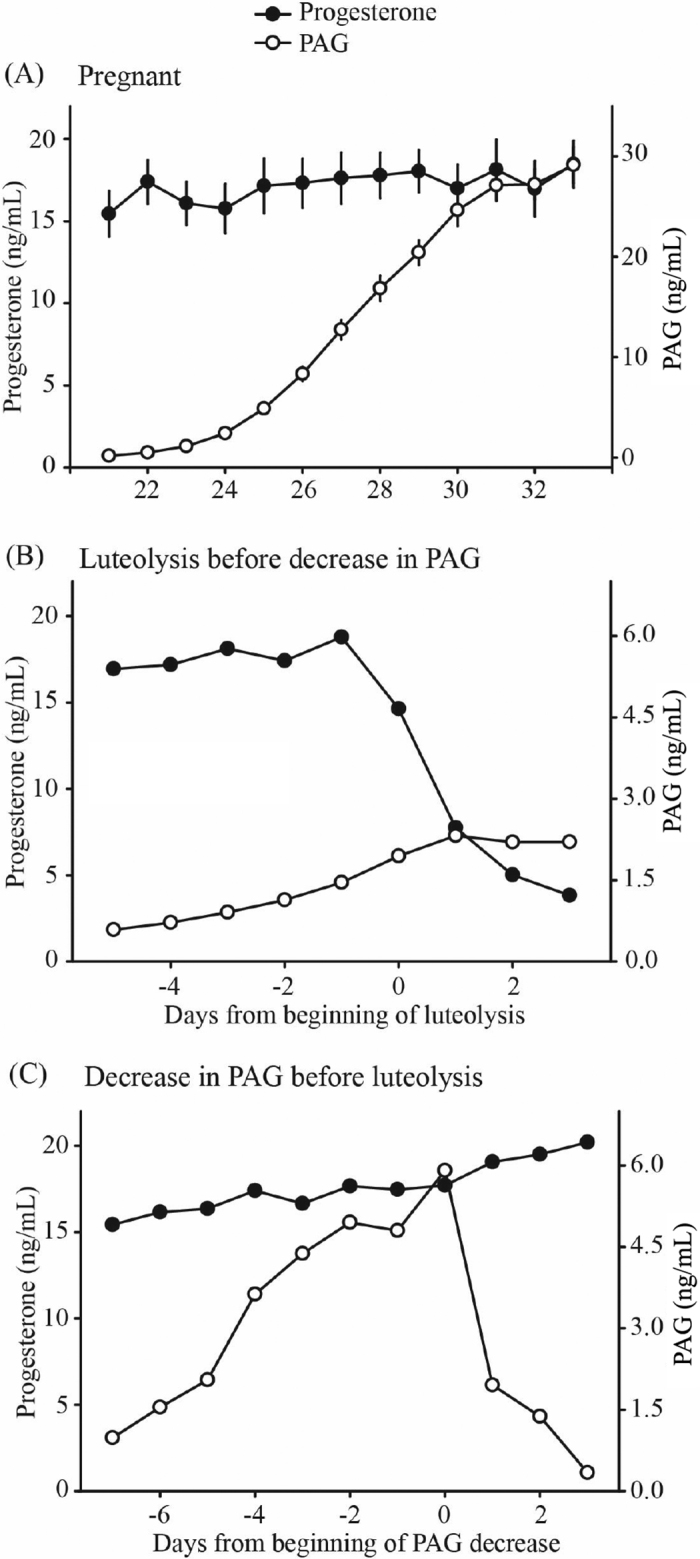
Concentrations of progesterone and pregnancy-associated glycoprotein (PAG) in cows that (A) maintained pregnancy or had pregnancy loss between d 20 and 33 with (B) luteolysis before decrease in PAG and (C) PAG decrease before luteolysis. For cows with pregnancy loss, data were normalized to the onset of luteolysis or decrease in PAG. When luteolysis occurred before decrease in PAG, the mean day of luteolysis (d 0) was d 27.7 ± 1.5; n = 8. When PAG decreased before luteolysis, the mean day of onset of PAG decrease (d 0) was d 29.3 ± 1.5; n = 7). Note the differences in scale for concentrations of PAG for cows that were pregnant and cows that had pregnancy loss. SEM were omitted in panels B and C.

Based on ultrasound evaluation, pregnancy loss in heifers occurs either when luteolysis precedes embryonic death or when embryonic death precedes luteolysis (Kastelic et al., 1991). Nevertheless, the incidence of pregnancy loss reported was 3%, much lower than the current rates of pregnancy loss in high-producing dairy cows (Santos et al., 2004; Wiltbank et al., 2016b, 2018). Other studies have attempted to investigate the cause of spontaneous and induced pregnancy loss in heifers and lactating cows and also reported embryonic death before luteolysis and luteolysis before embryonic death (Humblot et al., 1988; Giordano et al., 2012; Gábor et al., 2016). The novelty of our study is that we investigated spontaneous pregnancy loss in high-producing dairy cows using daily assessment of P4 and PAG, allowing detailed investigation of the onset of either luteolysis or conceptus failure (decrease in PAG). A limitation in multiple studies has been the diagnosis of early pregnancy to allow detection of early pregnancy loss (Ealy and Seekford, 2019; Szenci, 2021). Importantly, we were able to detect and investigate pregnancy loss as early as d 20 after GnRH, giving new insight into the frequency and causes (luteal vs. conceptus failure) of pregnancy loss during this period in the first month of pregnancy.

The second hypothesis—that pregnancy loss in high-producing dairy cows occurs due to luteal failure in some cows but due to failure of the conceptus before luteal regression in other cows—was supported. We were unable to detect pregnancy loss in which luteolysis and decrease in PAG occurred at the same time. Indeed, about 50% of pregnancy loss occurred in each scenario: luteal versus conceptus failure. As previously demonstrated by manipulative studies that either regressed the CL (PGF_2α_ treatment) or killed the embryo (hypertonic saline), both luteal demise and embryonic mortality can lead to pregnancy loss in cattle (Giordano et al., 2012). Interestingly, concentrations of PAG did not change for at least 3 d after the beginning of luteal regression in the present study, in contrast to previous studies in which PAG/PSPB decreased 1 to 2 d after PGF_2α_ treatment (Semambo et al., 1992; Giordano et al., 2012). The variability in the temporal changes in PAG after pregnancy loss in different studies might be explained by measurement of different PAGs, induction of pregnancy loss at different times (d 39 in Giordano et al., 2012), a difference in natural (this study) versus induced pregnancy loss, or parity (Middleton et al., 2022). In contrast, the P4 profiles from this and other studies (Kastelic et al., 1991; Semambo et al., 1992; Giordano et al., 2012) are similar for pregnancy loss that occurred due to embryonic death (PAG decrease before luteolysis), with none of the studies reporting a rapid luteal regression. Instead, changes in P4 have been reported to occur 3 to 42 d after cessation of embryonic heartbeat.

An intriguing aspect of pregnancy loss, highlighted in this study, is related to the occurrence of luteal regression in pregnant animals after the classic period of IFN-τ–mediated luteal maintenance (d 17 to 22). This could be related to the more variable and later luteolysis that occurs in some dairy cows compared with heifers (Domingues et al., 2020). In a recent report (Cunha et al., 2022), 37% of nonbred dairy cows had atypical estrous cycles, and the onset of luteolysis ranged from d 13 to 34 of the estrous cycle. Potentially of greater import, there appears to be a change in the mechanisms that maintain the CL of pregnancy after d 25, as the uterus begins to respond to oxytocin, whereas secreted PGF_2α_ does not appear to reach the CL to induce luteolysis (Wiltbank et al., 2018; Drum et al., 2020). Further studies are needed to elucidate the mechanisms that maintain the CL or allow CL regression during this critical period.

Pregnancy loss between d 33 and 61 occurred in 12.5% of cows (5/40; 3 control and 2 hCG). Concentrations of PAG on d 33 were not different between cows that underwent pregnancy loss and those that maintained pregnancy until d 61 (23.9 ± 2.1 vs. 29.1 ± 1.8, respectively; *P* = 0.32). In 4 cows, pregnancy loss occurred by d 47; in 1 cow, the loss occurred between d 47 and 61.

In conclusion, hCG treatments on d 7 and 13 increased circulating P4 but this was not associated with earlier embryonic attachment, as measured by PAG, or with increased concentrations of PAG during the first month of gestation. Nevertheless, PAG was increased in the hCG group on d 47 and 61. Additionally, there was high pregnancy loss from d 20 to 33 of pregnancy, with ~50% of these losses initiated by regression of the CL and ~50% initiated by death of the conceptus; that is, luteolysis occurred before a decrease in PAG or PAG decreased but luteolysis did not occur, respectively.
